# Longitudinal course of GDF15 levels before acute hospitalization and death in the general population

**DOI:** 10.1093/geroni/igab046.2513

**Published:** 2021-12-17

**Authors:** Juliette Tavenier, Ove Andersen, Jan O Nehlin, Janne Petersen

**Affiliations:** 1 Copenhagen University Hospital Hvidovre, Hvidovre, Hovedstaden, Denmark; 2 Copenhagen University Hospital Frederiksberg, Frederiksberg, Hovedstaden, Denmark

## Abstract

Growth differentiation 15 (GDF15) is a potential novel biomarker of biological aging. To separate the effects of chronological age and birth cohort from biological age, longitudinal studies investigating associations of GDF15 levels with adverse health outcomes are needed. We investigated changes in GDF15 levels over 10 years in an age-stratified sample of the general population and their relation to the risk of acute hospitalization and death. Serum levels of GDF15 were measured three times in 5-year intervals in 2176 participants aged 30, 40, 50, or 60 years from the Danish population-based DAN-MONICA cohort. We assessed the association of single and repeated GDF15 measurements with the risk of non-traumatic acute hospitalizations. We tested whether changes in GDF15 levels over 10 years differed according to the frequency of hospitalizations within 2 years, or survival within 20 years, after the last GDF15 measurement. The change in GDF15 levels over time was dependent on age and sex. Higher GDF15 levels and a greater increase in GDF15 levels were associated with an increased risk of acute hospitalization in adjusted Cox regression analyses. Participants with more frequent admissions within 2 years, and those who died within 20 years, after the last GDF15 measurement already had elevated GDF15 levels at baseline and experienced greater increases in GDF15 levels during the study. The change in GDF15 levels was associated with changes in C-reactive protein and biomarkers of kidney, liver, and cardiac function. Monitoring of GDF15 starting in middle-age could be valuable for the prediction of adverse health outcomes.

